# Machine Learning Estimation of Gestational Age at Delivery Using Linked Mother–Infant Electronic Health Records Across Two Health Systems

**DOI:** 10.1002/pds.70516

**Published:** 2026-10-29

**Authors:** Cosmin A. Bejan, Xiaotong Yang, Amelie Pham, Layth Qassem, Abin Abraham, Leena Choi, S. Trent Rosenbloom, Lana X. Garmire, Elizabeth J. Phillips

**Affiliations:** ^1^ Department of Biomedical Informatics Vanderbilt University Medical Center Nashville Tennessee USA; ^2^ Department of Computational Medicine and Bioinformatics University of Michigan Ann Arbor Michigan USA; ^3^ Division of Maternal Fetal Medicine, Department of Obstetrics and Gynecology Vanderbilt University Medical Center Nashville Tennessee USA; ^4^ Division of Neonatology Children's Hospital of Philadelphia Philadelphia Pennsylvania USA; ^5^ Department of Biostatistics Vanderbilt University Medical Center Nashville Tennessee USA; ^6^ Department of Medicine Vanderbilt University Medical Center Nashville Tennessee USA

**Keywords:** electronic health records, gestational age estimation, gradient boosting, machine learning, random forest

## Abstract

**Purpose:**

This study aimed to develop and evaluate machine‐learning models for estimating gestational age at delivery using EHR data from two health systems.

**Methods:**

We trained random forest, gradient boosting, and ensemble models using EHR data from mother–infant dyads at Vanderbilt University Medical Center (VUMC) and independently reimplemented and retrained the modeling pipeline at the University of Michigan (UMich). We further analyzed EHR predictors of gestational age, assessed temporal drift in EHR data elements, and evaluated model performance stratified by gestational‐age category at delivery.

**Results:**

The study included pregnancies corresponding to 54 344 and 34 345 mother–infant dyads at VUMC (2005–2025) and UMich (2012–2024), respectively. The gestational age predictions of the ensemble models showed high agreement with the reference standard on the VUMC dataset (±1 week: 85.1%, ±2 weeks: 94.3%, MAE: 4.4 days). When the same modeling pipeline was independently implemented and retrained on the UMich dataset, the ensemble models achieved higher agreement with the reference standard (±1 week: 93.1%, ±2 weeks: 97.8%, MAE: 2.8 days). Further, performance was better among pregnancies delivered in more recent years, and among full‐ and late‐term deliveries compared with preterm deliveries.

**Discussion:**

The results indicate that supervised machine learning methods leveraging linked mother–infant EHRs can accurately estimate gestational age at delivery overall, although performance was substantially lower for preterm deliveries. Reproducibility of the modeling approach and portability of the analytic workflow were demonstrated across the two health systems.

**Conclusion:**

This study presents a robust machine learning framework to estimate gestational age at delivery, whose development process was reproducible across two health systems. The framework has the potential to support large‐scale real‐world maternal and neonatal health studies requiring retrospective estimation of gestational age.

## Key Points

- This retrospective study developed and evaluated machine‐learning models to estimate gestational age at delivery using linked mother–infant EHR data from two health systems.
- A supervised machine learning framework accurately estimated gestational age at delivery at both sites after site‐specific model training, supporting reproducibility of the approach and portability of the analytic workflow.
- The study findings support further evaluation of the modeling approach for retrospectively estimating gestational age in real‐world data sources with incomplete gestational‐age documentation.

## Plain Language Summary

Knowing how long a pregnancy lasted at the time of delivery (gestational age) is essential for maternal and infant health research. However, this information is often missing or incomplete in electronic health records (EHRs). In this study, we developed and evaluated machine learning models to estimate gestational age at delivery using routinely collected EHR data from linked mother–infant pairs across two large health systems. The models showed high agreement with documented clinical gestational‐age estimates, with predictions typically within 1–2 weeks of the reference value and performed well after site‐specific training at both health systems. Performance was highest for more recent pregnancies and full‐term births and lower for preterm deliveries. Overall, these findings demonstrate that machine learning can estimate gestational age from EHR data with good accuracy for most births, strengthening pharmacoepidemiologic studies of drug safety during pregnancy and supporting future maternal and infant health research.

## 1 Introduction

Accurate estimation of gestational age is fundamental to pregnancy and offspring health outcome research. In pharmacoepidemiologic studies of drug safety in pregnancy, for example, accurate definition of pregnancy onset and exposure windows is particularly critical because fetal susceptibility to pharmacologic agents and teratogenic risk vary by developmental stage [1, 2, 3]. Accurate ascertainment of pregnancy timing is also important for evaluating medication use when pregnancy is a contraindication, is addressed in product labeling, or is included in risk‐minimization measures. However, errors in dating pregnancy onset are common and may occur for numerous reasons, including provider data entry errors, lack of ultrasound documentation, incomplete records when patients transfer care in pregnancy, or inadequate system infrastructure. Such inaccuracies in pregnancy dating can lead to misclassification of drug exposures, introducing bias and undermining the validity of study findings [4, 5, 6, 7, 8, 9]. These challenges are particularly relevant to studies using electronic health record (EHR) and administrative claims databases, because observational studies based on these data sources are a primary method for evaluating the safety and effectiveness of treatments in pregnant populations who are typically excluded from randomized clinical trials due to design challenges and ethical concerns [10, 11, 12]. In these data sources, gestational age estimates are often missing, incomplete, or incorrectly recorded. Such gaps may undermine statistical power in perinatal and pharmacoepidemiologic research involving pregnant populations, including drug‐wide association studies [13, 14, 15], and may also introduce bias if missingness is related to confounding factors.

Existing approaches to gestational‐age estimation have been developed mainly in US administrative claims data, and few have used EHR data with linked maternal and infant records [16, 17, 18, 19]. The objectives of this study were to: (1) develop and internally evaluate machine learning algorithms for estimating gestational age at live birth using linked maternal–infant EHR data from Vanderbilt University Medical Center (VUMC); (2) independently reimplement and retrain the same modeling pipeline using EHR data from the University of Michigan (UMich) to assess reproducibility of the model‐development approach and portability of the analytic workflow; (3) assess the contribution of maternal, EHR‐specific, and pediatric predictors to model performance; (4) evaluate the impact of temporal drift in EHR data elements on model performance; and (5) assess performance stratified by gestational‐age category at delivery.

## 2 Methods

This is a retrospective prediction‐model development and evaluation study using routinely collected linked mother–infant EHR data from Vanderbilt University Medical Center (VUMC) and the University of Michigan (UMich) Medicine Healthcare System. The study involved the development and validation of machine learning algorithms to estimate gestational age at live birth for each pregnancy that has mother–infant dyad data. All machine learning models were initially trained on the VUMC dataset and independently retrained using the same modeling pipeline on the UMich dataset to assess the robustness and replicability of the development process. The study was approved with waiver of consent by the Institutional Review Board (IRB) at VUMC (IRB# 231322) and UMich (HUM# 00168171). The study follows the TRIPOD+AI guideline for transparent reporting of clinical prediction models developed using machine learning methods [20]. (Table S1).

### 2.1 Data Sources

Clinical data at VUMC were extracted from the Research Derivative (RD), a research‐focused repository of the institution's EHR system. The RD contains records on over 5.5 million patients, with preexisting mother–infant linkages available since 2005. To replicate the study, we used EHR data from the UMich database, which includes clinical records for approximately 5 million patients dating back to 2006. The mother–infant linkages were established and maintained by the respective institutional data resources prior to this study and did not involve a record‐linkage algorithm developed or applied by the study team. Details of the procedures used to establish the preexisting mother–infant linkages and the proportion of mother–infant pairs successfully linked at each institution were not available to the study team. Specific data elements extracted from both EHR systems include demographic data, encounter information, clinical estimates of gestational age, APGAR scores, birth weight, and disease diagnoses based on the International Classification of Diseases, 9th/10th Revision, Clinical Modification (ICD‐9/10‐CM) billing codes.

### 2.2 Study Population

The initial study population included all mother–infant dyads from the two EHR databases. To address within‐mother correlation and potential bias, one infant per mother was randomly selected for mothers with multiple pregnancies or multiple births, resulting in approximately independent mother–infant dyads for model training. Further, to reduce potential sources of bias in the data, mothers who had inadequate prenatal care or pregnancies that did not result in a live birth were also excluded. Adequate prenatal care was defined using ICD diagnostic codes (Table S2) and the requirement of at least two prenatal encounters. Live births were identified using ICD codes documented at the time of delivery (Table S3). In summary, the final dataset used for algorithm development and validation consisted of unique mother–infant dyads with pregnancies resulting in a live birth, in which mothers had adequate prenatal care and a documented clinical estimate of gestational age at delivery, as described below.

### 2.3 EHR‐Based Extraction of Gestational Age Estimates

Clinical gestational age (CGA) estimate at delivery was extracted from each maternal record and used as the reference standard outcome for training and validating the machine learning models. The CGA estimates represent the birth attendant's final assessment of the infant's gestational age. The underlying clinical dating method could not be determined from the available records at either institution. At VUMC, the CGA estimates are recorded as textual patterns indicating weeks + days of gestation (e.g., “39 weeks 2/7” corresponds to 275 gestational days; more examples are provided in Table S4). All these textual expressions were transformed into a numeric representation of gestational age in days using regular expressions. Cases with no CGA expression within 1 day of delivery, or with expressions that could not be parsed (see examples in Table S5), were marked as missing or invalid, respectively.

### 2.4 Algorithm‐Based Estimation of Gestational Age

We trained supervised machine learning models on mother–infant dyad data to predict the gestational age as a continuous numerical outcome. Specifically, to solve this regression task, we used random forest (RF), gradient boosting (GB), and an ensemble approach that involved averaging the predictions from the former two algorithms. First, we randomly split the mother–infant dyads into training (80%) and test (20%) sets. We then trained machine learning models and optimized hyperparameters using fivefold cross‐validation on the training set. Table S6 summarizes the hyperparameters examined, the search ranges used, and the optimal values identified. We used a quantile transformation on the CGA values to address their left‐skewed distribution and reduce the influence of outliers, which enhanced the performance of our regression models. Finally, we retrained the models on the full training set with the best‐performing hyperparameter values identified through grid search and evaluated the retrained models on the test set. To provide a point of comparison for the machine learning models, we also implemented simple constant‐value baselines using the mean and median CGA values from the training set.

Candidate predictors were selected according to their availability in the EHR data and clinical relevance to pregnancy and delivery. For each mother–infant dyad, predictors used for model training were derived from three data sources: (1) pregnancy‐related data from maternal records, (2) standard data elements commonly stored in EHR systems, and (3) pregnancy‐related data from infant records. The EHR‐specific predictors included the infant birth weight, the 1 and 5‐min APGAR scores, the number of previous pregnancies, and the number of fetuses in the current pregnancy associated with the mother–infant dyad. We addressed missing APGAR scores and infant birth weight values by imputing them with the respective median values calculated from the training set and applying these values to the corresponding test set. If multiple APGAR or birth weight values were recorded for the same infant, we selected the maximum value for each predictor. For the predictors extracted from maternal records, we used ICD codes indicating preterm birth (Table S7), term or postterm birth (Table S8), fetal growth restriction (Table S9), and excessive fetal growth (Table S10). Similarly, from infant records, we extracted ICD‐based predictors denoting preterm birth (Table S11), term or postterm birth (Table S12), small for gestational age (Table S13), and large for gestational age (Table S14). We also extracted predictors related to the demographic characteristics of the mother–infant dyad, such as maternal age at delivery, maternal race and ethnicity, infant sex, and infant race and ethnicity. Following a pilot study on the training set, where ICD‐based predictors were encoded as either binary or count variables at the individual code or broader category level, we determined that representing ICD codes by the number of distinct days or visits on which they were assigned during pregnancy was the most effective approach. To extract the ICD‐based predictors for each mother–infant dyad, we estimated the pregnancy duration based on the delivery date (corresponding to the infant's date of birth) and the median CGA value from the training set. Use of the dyad‐specific CGA was avoided to prevent target leakage. Lastly, to evaluate the contribution of different predictor categories, we trained models using three configurations: (C1: Maternal), incorporating only predictors derived from maternal records; (C2: Maternal+EHR), adding EHR‐derived features; and (C3: Maternal+EHR + Pediatric), including all available predictors. C1 and C2 correspond to data environments ranging from administrative databases to EHR systems without mother–infant linkage, while C3 represents environments with linked mother–infant data, which are more difficult to obtain in practice. Table S15 summarizes the predictors used for each configuration.

### 2.5 Evaluation

We assessed algorithm performance over the test sets using the proportion of pregnancies with algorithm‐based gestational age (AGA) estimation within ±1 week (±7 days) and ±2 weeks (±14 days) of the reference standard, and mean absolute error (MAE) between AGA and CGA estimates. To estimate the 95% confidence intervals (CIs) for these performance metrics, we applied bootstrapping by generating 1000 resampled test sets with replacement, computing the metrics for each, and extracting the 2.5th and 97.5th percentiles of the resulting empirical distribution [21]. Further, we investigated potential data drift arising from temporal shifts in ICD coding and billing practices by stratifying the results for pregnancies with delivery dates occurring before or on/after October 1, 2015, the date when the transition from ICD‐9‐CM to ICD‐10‐CM officially took place. Finally, we assessed variations in algorithm performance by delivery‐status category, stratifying the results into preterm (less than 37 weeks of gestation), early term (37 0/7 weeks of gestation through 38 6/7 weeks of gestation), full term (39 0/7 weeks of gestation through 40 6/7 weeks of gestation), late term (41 0/7 weeks of gestation through 41 6/7 weeks of gestation), and postterm (42 0/7 weeks of gestation and beyond) categories [22].

### 2.6 Method Replication

To assess the robustness and reproducibility of our method development and determine whether comparable performance could be achieved, we independently replicated the machine learning framework for gestational age estimation on the UMich dataset. This replication used identical inclusion and exclusion criteria, predictor variables, and model training and evaluation procedures. Notably, because the machine learning models were retrained at UMich, this analysis evaluated cross‐site reproducibility of the development process and portability of the workflow, rather than transportability of the VUMC‐fitted models.

## 3 Results

### 3.1 Selection of Mother–Infant Dyads

The initial VUMC and UMich study populations comprised 91 471 and 55 733 mother–infant dyads, corresponding to 68 659 and 39 969 mothers, respectively (Figure 1). The delivery years for dyads at VUMC and UMich spanned 2005–2025 and 2012–2024, respectively. During these periods, missing or invalid CGA values were most common in the early years following implementation of mother–infant linkages in both EHR systems (Figure 2a,b). After retaining one infant per mother and excluding non‐live births and mothers with insufficient prenatal care, the final analytic datasets included 60 057 (65.7%) mother–infant dyads at VUMC and 37 861 (67.9%) at UMich. Dyads with valid CGA values in VUMC (*N* = 54 344) and UMich (*N* = 34 345) datasets were subsequently used for algorithm development (Figure 1). The average maternal age was 28.9 years in the VUMC dataset and 31.8 years in the UMich dataset (Table 1). Both datasets had predominantly White mothers and a slightly higher proportion of male infants. The CGA distributions were comparable, each with a median of 274 gestational days (Table 1, Figure 2c,d).

**FIGURE 1:**
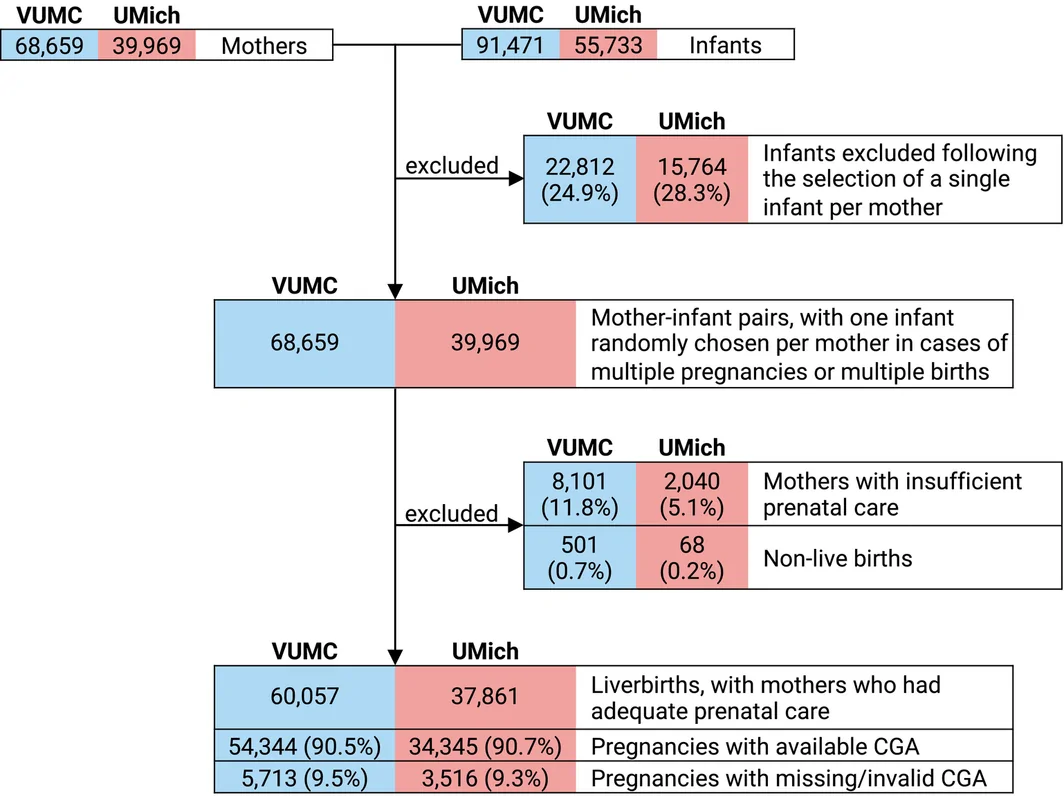
Selection of mother–infant dyads for algorithm‐based estimation of gestational age in the electronic health record (EHR) systems of Vanderbilt University Medical Center (VUMC) and the University of Michigan (UMich) Medicine Healthcare System. CGA, clinical estimate of gestational age.

**FIGURE 2:**
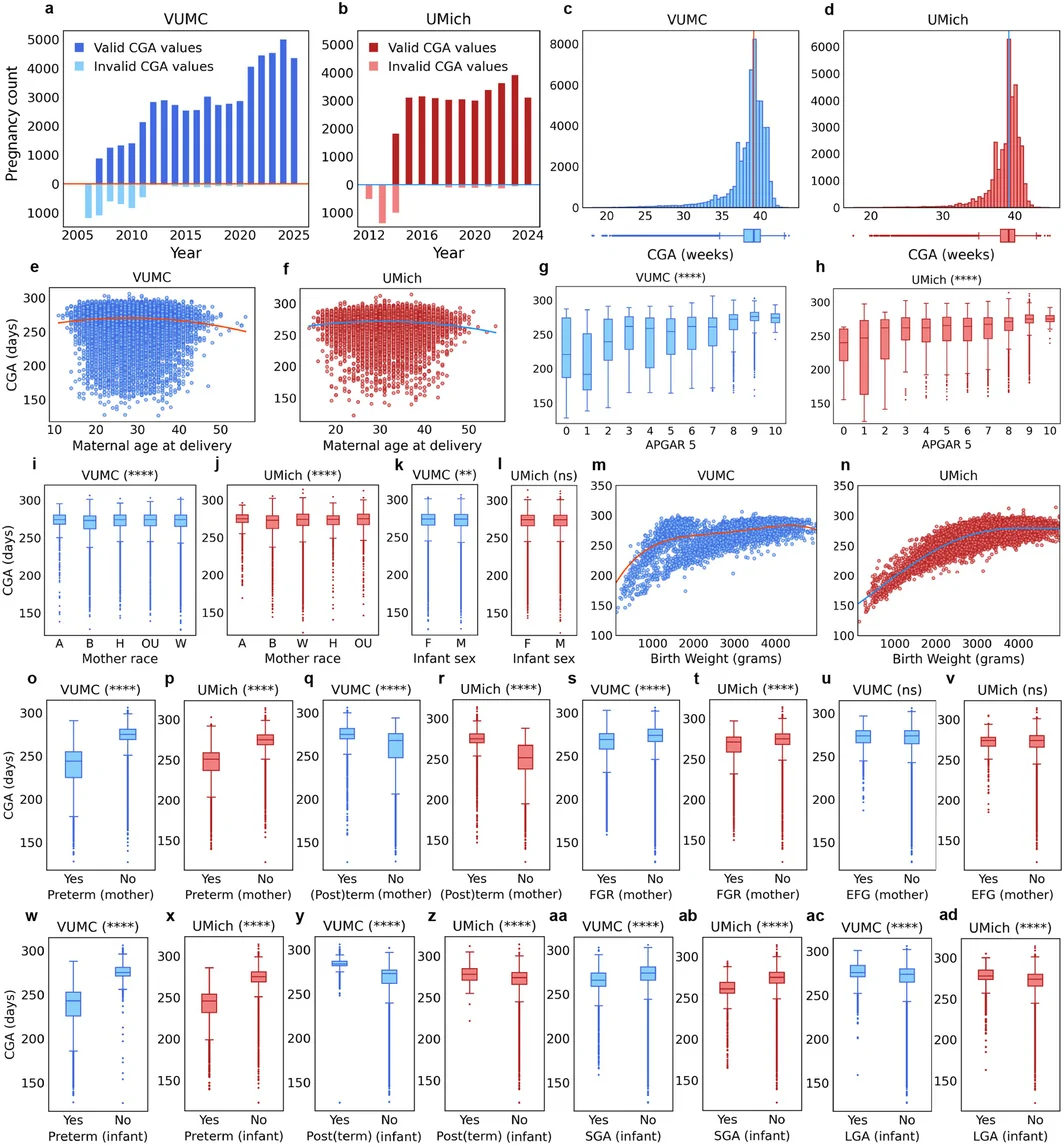
Comparative analysis of clinical gestational age (CGA) estimates across VUMC and UMich datasets. (a, b) Pregnancies with valid and invalid CGA estimates, by delivery year. (c, d) CGA distributions. The median CGA in both datasets is 274 days (39 1/7 weeks). (e, f) Maternal age at delivery and CGA. A scatterplot with quadratic fit was used to illustrate the nonlinear trend. (g, h) APGAR score at 5 min after delivery and CGA. (i, j) Maternal race and CGA. (k, l) Infant sex and CGA. (m, n) Birth weight and CGA. A smooth quartic spline was employed to visualize the trend. (o, p) Preterm in maternal records and CGA. (q, r) Term/postterm in maternal records and CGA. (s, t) Fetal growth restriction (FGR) in maternal records and CGA. (u, v) Excessive fetal growth (EFG) in maternal records and CGA. (w, x) Preterm in infant records and CGA. (y, z) Term/postterm in infant records and CGA. (aa, ab) Small for gestational age (SGA) in infant records and CGA. (ac, ad) Large for gestational age (LGA) in infant records and CGA. Predictor analysis (e, ad) was conducted using the dyads from the training set. Differences in CGA distributions were assessed using the Mann–Whitney U test for binary predictors and the Kruskal–Wallis H test for multicategorical predictors. Statistical significance levels are shown in the plot titles using the following notation: ‘****’ for *p* ≤ 0.0001, ‘***’ for 0.0001 < *p* ≤ 0.001, ‘**’ for 0.001 < *p* ≤ 0.01, ‘*’ for 0.01 < *p* ≤ 0.05, and ‘ns’ for *p* > 0.05. Given the large sample sizes, *p* values should be interpreted alongside the magnitude of observed differences and not as measures of clinical importance.

**TABLE 1:** Characteristics of mother–infant dyads from VUMC and UMich datasets.

Characteristic	VUMC	UMich
Mother–infant dyads, *N*	54 344	34 345
Maternal age at delivery, mean years (SD)	28.9 (5.8)	31.8 (5.3)
Mother race/ethnicity, *N* (%)		
White	30 546 (56.2%)	23 575 (68.6%)
Black	7648 (14.1%)	4146 (12.1%)
Hispanic	8387 (15.4%)	1984 (5.8%)
Asian	2101 (3.9%)	3130 (9.1%)
Other/unknown	5662 (10.4%)	1510 (4.4%)
Infant sex, *N* (%)		
Male	27 965 (51.5%)	17 584 (51.2%)
Female	26 379 (48.5%)	16 761 (48.8%)
CGA estimates in days		
Mean (SD)	269.5 (18.8)	270.9 (16.3)
Median	274	274
IQR	265.0–280.0	266.0–280.0
Min–Max	127.0–306.0	123.0–314.0
Delivery status, *N* (%)		
Preterm	7795 (14.3%)	4336 (12.6%)
Early term	14 090 (25.9%)	8874 (25.8%)
Full term	27 881 (51.3%)	18 333 (53.3%)
Late term	4350 (8.0%)	2644 (7.7%)
Postterm	228 (0.4%)	158 (0.5%)

Abbreviations: CGA, clinical gestational age; IQR, interquartile range; SD, standard deviation.

### 3.2 Predictor Analysis in Association With CGA

A visual analysis highlighting differences in gestational age trends across selected predictors is shown in Figure 2e,ad. The analysis was conducted using all mother–infant dyads from the VUMC (*N* = 43 475) and UMich (*N* = 27 476) training sets. Figure 2e,f indicate lower gestational age among young mothers and mothers of advanced maternal age in both datasets. Figure 2g,h demonstrate that higher 5‐min APGAR scores correspond to longer gestational ages among pregnancies with available APGAR scores (42% of pregnancies). Figure 2i,j show variations in gestational age across maternal race/ethnicity groups at both sites (*p* ≤ 0.0001). The highest mean gestational age was observed among Asian mothers and the lowest among Black mothers (VUMC: 271.7 versus 267.2 days; UMich: 273.0 versus 268.1 days), corresponding to absolute differences of 4.5 and 4.9 days, respectively. At VUMC, mean gestational age was slightly higher for mothers of female infants than for mothers of male infants (269.8 versus 269.2 days; *p* < 0.01). However, the absolute difference was only 0.6 days and is unlikely to be clinically meaningful. No difference by infant sex was observed at UMich (Figure 2k,l). Among pregnancies with available birth weight data (25% of pregnancies), gestational age showed a pronounced decrease within the first quartile of the birth weight distribution (Figure 2m,n). As expected, dyads in which both maternal and infant records contain preterm ICD codes exhibited significantly lower gestational ages (Figure 2o,p; 2w–x), whereas those with term or postterm codes show significantly higher gestational ages (Figure 2q,r; 2y–z). In both datasets, gestational age was significantly lower for mothers with fetal growth restriction (FGR) ICD codes (VUMC: 262.3 vs. 270.6 days; UMich: 264.5 vs. 272.3 days; *p* ≤ 0.0001) but did not differ significantly for those with excessive fetal growth (EFG) ICD codes (Figure 2s–v). Finally, Figure 2aa–ad indicate that gestational age was significantly lower for pregnancies with small for gestational age (SGA) ICD codes in infant records (VUMC: 262.7 vs. 270.0 days; UMich: 259.0 vs. 270.6 days; *p* ≤ 0.0001) and higher for those with large for gestational age (LGA) ICD codes (VUMC: 275.3 vs. 269.1 days; UMich: 276.8 vs. 270.6 days; *p* ≤ 0.0001).

### 3.3 CGA Estimation Performance

The results describing the performance of the machine learning algorithms for estimating gestational age on VUMC and UMich test sets are summarized in Table 2 and Figure 3. When evaluated on the VUMC test set with the full set of predictors (C3), the algorithms correctly classified 84.0%–85.2% of pregnancies within 1 week and 93.7%–94.3% within 2 weeks of the reference standard. Retraining the algorithms on the UMich dataset using the same predictors yielded higher agreement with the reference standard, with 91.1%–93.1% of estimates within 1 week and 97.0%–97.8% within 2 weeks. Across both test sets, the machine learning algorithms substantially outperformed the baseline estimates based on the mean and median gestational ages from the training sets (VUMC, ±1 week: 41.5%–51.7%, ±2 weeks: 72.4%–80%; UMich, ±1 week: 42.7%–53%, ±2 weeks: 75.1%–81.6%). On the VUMC test set, the machine learning algorithms achieved MAE values ranging from 4.4 to 4.6 days, representing improvements of 6.8 to 7.9 days compared with the mean (12.3 days) and median (11.4 days) baselines. On the UMich test set, MAE values ranged from 2.8 to 3.1 days, corresponding to improvements of 7.3–8.2 days relative to these baselines (Table 2). Overall, the ensemble method achieved the best performance, followed in close succession by random forest and gradient boosting (Table 2, Figure 3a,b). The predicted gestational ages from the three algorithms align closely with the reference estimates, as evidenced by substantial overlap in their distributions depicted in Figure 3e,f,i,j,m,n and the strong agreement shown in the scatterplots from Figure 3g,h,k,l,o,p, with Pearson correlation coefficients ranging from 0.86 to 0.89 for VUMC and 0.94 to 0.95 for UMich data. Across the three predictor configurations, model performance improves with each addition of data elements, from C1: maternal predictors alone to C2: maternal and EHR, and, finally, C3: maternal, EHR, and pediatric predictors (Figure 3c,d, Table S16). The temporally stratified analysis indicates that overall model performance is improved for pregnancies delivered on or after October 1, 2015, compared with those delivered before (Table S17). Finally, the stratified analysis by delivery status shows that the models perform best for full‐term and late‐term deliveries, but substantially worse for preterm deliveries (Table S18).

**TABLE 2:** Algorithm performance evaluation on the VUMC and UMich test sets.

Dataset	Dyads	Algorithm	% of AGA estimates within 1 week (95% CI)	% of AGA estimates within 2 weeks (95% CI)	MAE (95% CI)
VUMC	10 869	Mean	41.5 (40.5–42.4)	72.4 (71.5–73.3)	12.3 (12.0–12.6)
		Median	51.7 (50.8–52.6)	80.0 (79.3–80.8)	11.4 (11.1–11.7)
		Random Forest	85.2 (84.5–85.9)	93.9 (93.5–94.4)	4.4 (4.2–4.5)
		Gradient Boosting	84.0 (83.3–84.6)	93.7 (93.3–94.2)	4.6 (4.5–4.8)
		Ensemble	85.1 (84.4–85.8)	94.3 (93.9–94.8)	4.4 (4.2–4.5)
UMich	6869	Mean	42.7 (41.6–43.9)	75.1 (74.1–76.2)	11.0 (10.7–11.3)
		Median	53.0 (51.8–54.2)	81.6 (80.7–82.5)	10.4 (10.1–10.7)
		Random Forest	92.9 (92.2–93.4)	97.5 (97.1–97.8)	2.8 (2.7–2.9)
		Gradient Boosting	91.1 (90.4–91.8)	97.0 (96.6–97.4)	3.1 (3.0–3.2)
		Ensemble	93.1 (92.5–93.7)	97.8 (97.5–98.2)	2.8 (2.7–2.9)

Abbreviations: AGA, algorithm‐derived gestational age; CI, confidence interval; MAE, mean absolute error.

**FIGURE 3:**
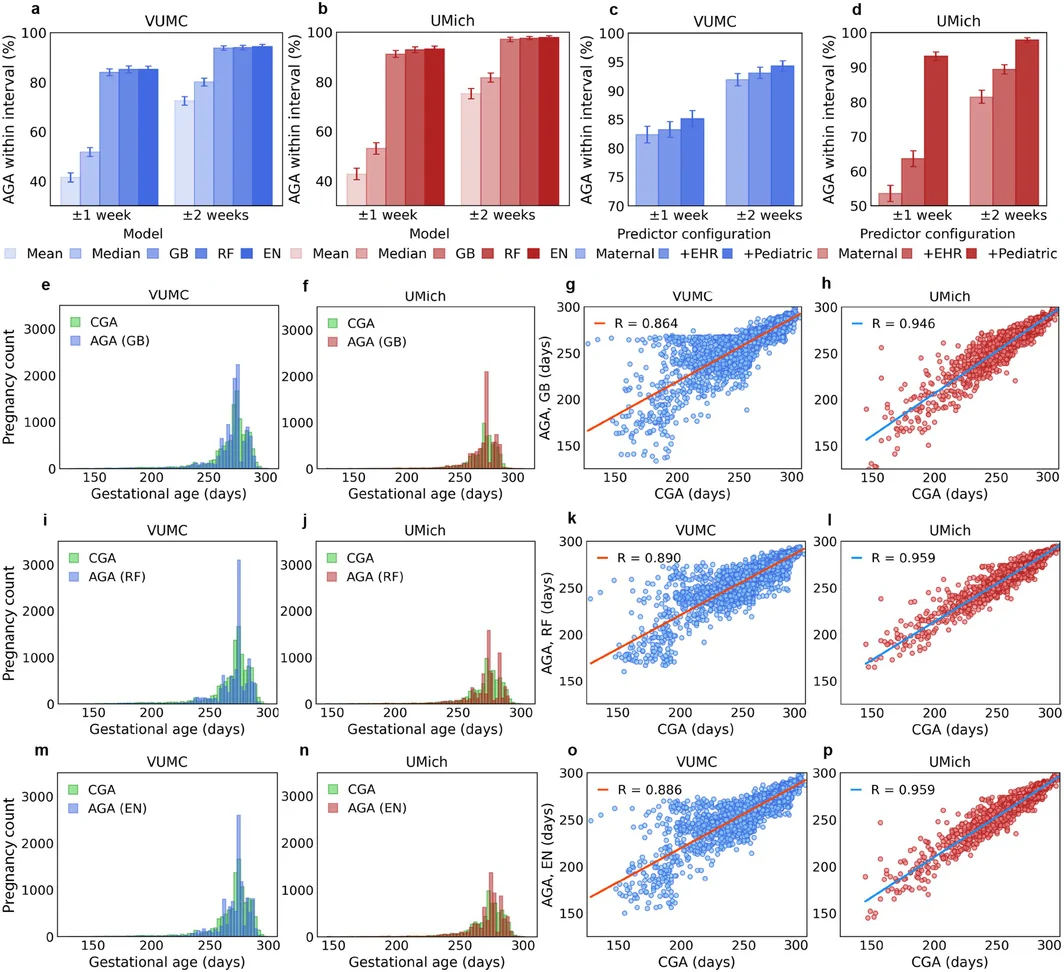
Performance evaluation of methods for gestational age prediction. (a, b) Comparative performance of baselines (mean and median) and machine learning methods measured by the proportion of gestational age predictions within 1 and 2 weeks of the reference standard. (c, d) Comparison of ensemble method performance using three predictor configurations: maternal only, maternal + EHR‐specific, and maternal + pediatric + EHR‐specific predictors. (e, f) Comparison of gestational age distributions between the reference standard and gradient boosting predictions. (g, h) Scatterplot of reference standard versus gradient boosting predictions. (i, j) Comparison of gestational age distributions between the reference standard and random forest predictions. (k, l) Scatterplot of reference standard versus random forest predictions. (m, n) Comparison of gestational age distributions between the reference standard and ensemble method predictions. (o, p) Scatterplot of reference standard versus ensemble method predictions. AGA, algorithm‐derived gestational age; CGA, clinical gestational age; EN, ensemble method; GB, gradient boosting; R, Pearson correlation coefficient; RF, random forest.

## 4 Discussion

Using EHR data from mother–infant dyads, we developed and evaluated machine‐learning models for estimating gestational age at delivery. We independently reimplemented and retrained the same modeling pipeline using data from a separate health system. This cross‐site replication supports the reproducibility of the modeling approach and the portability of the analytic workflow.

Based on our review of the published literature, most prior gestational‐age estimation methods have used administrative claims data, including methods that assume a uniform pregnancy duration and rule‐based or machine learning models based largely on ICD code‐derived predictors [16, 17, 18, 19, 23, 24, 25, 26, 27, 28]. Most were developed using pregnancies ending in live birth, with few addressing pregnancies ending in stillbirth, spontaneous abortion, or termination. The study most closely related to ours is by Zhu et al. [25], who used claims data from 114 117 pregnancies associated with mother–infant dyads in the Medicaid Analytic eXtract (MAX) to develop machine learning methods for estimating gestational age at live birth. Their best‐performing model was an ensemble learning approach, Super Learner [29], which combined predictions from LASSO regression [30], Bayesian additive regression trees (BART) [31], extreme gradient boosting [32], neural networks, Bayesian generalized linear models, generalized linear regression, and random forests, achieving 85.1% and 96.4% of estimates within 1 and 2 weeks of the reference standard, respectively. In comparison with prior administrative claims‐based studies, including Zhu et al., our best‐performing EHR‐based approach demonstrated comparable or improved performance, achieving agreement within 1 and 2 weeks of 85.1% and 94.3% at VUMC and 93.1% and 97.8% at UMich.

This study exhibits several notable strengths. First, we conducted an analysis of gestational age patterns in relation to multiple EHR‐derived predictors. This analysis revealed an inverted U‐shaped relationship between gestational age and maternal age, consistent with prior literature describing similar [33, 34] or related U‐shaped associations between maternal age and preterm birth risk [35, 36, 37, 38]. Additional findings corroborated by previous studies include associations between prematurity, defined as delivery before 37 weeks of gestation, low birth weight [39, 40], small for gestational age [41, 42], and fetal growth restriction [43, 44, 45]. Second, we developed several methodological configurations that account for differences in predictor availability and mother–infant linkage, thereby supporting method implementation across diverse data environments, including administrative claims databases and EHR systems. Third, the temporally stratified analysis indicated that ICD‐10‐CM predictors contributed to substantially improved performance compared with ICD‐9‐CM predictors. In addition, stratification by delivery status showed that the machine learning algorithms performed optimally for full‐term and late‐term deliveries but were moderate for preterm deliveries, consistent with findings reported in prior studies [16, 17, 19, 25]. Finally, independent reimplementation and site‐specific retraining of the machine learning framework at a second EHR site supported the reproducibility of the modeling approach and portability of the analytic workflow across the two health systems studied. Notably, the performance was improved in the UMich dataset, potentially due to its reduced reliance on ICD‐9‐CM predictors. In contrast, the VUMC dataset includes a substantial number of pregnancies from earlier years (i.e., 2005–2011), which may be associated with greater gestational age measurement error due to more limited ultrasound technology (e.g., lower‐resolution imaging) and less advanced modeling methodologies available at the time.

This study has several limitations that warrant further investigation and refinement in future research. Notably, the machine learning methods demonstrated substantially lower performance for preterm deliveries. This limitation is particularly important for pharmacoepidemiologic studies, in which gestational age is used to define etiologically relevant exposure windows. Misestimation of gestational age among preterm pregnancies could result in misclassification of the timing, duration, or trimester of medication exposure, potentially biasing estimates of medication safety. Thus, the current models should be applied cautiously in analyses that include or specifically focus on preterm deliveries. Another limitation is that birth weight and APGAR values were frequently missing in the EHR data and were imputed using medians calculated from the training set. Future refinements should evaluate more robust approaches for handling missing predictor data, such as multiple imputation using other predictors available at the time of gestational‐age estimation while excluding CGA estimates to prevent outcome leakage. Future research should also explore incorporating additional predictors derived from maternal and infant records, including a broader range of ICD codes, Current Procedural Terminology (CPT) codes, and Neonatal Intensive Care Unit (NICU)‐related data elements. Further, this study was restricted to linked mother–infant dyads resulting in live birth, as nearly all pregnancies in both VUMC and UMich study populations were associated with live‐birth ICD codes. Details of linkage procedures and success rates were unavailable at both sites, limiting assessment of linkage accuracy, completeness, and potential selection bias. Consequently, the approach was not evaluated in pregnancy settings where identification and dating may be most challenging: pregnancies that are ongoing or early‐stage at the time of data extraction and pregnancies ending in stillbirth, ectopic pregnancy, spontaneous abortion, or termination, for which child linkage may be unavailable. The relevance of this limitation will depend on the completeness and structure of available pregnancy data. Future work should evaluate outcome‐specific models and integrate approaches for early pregnancy identification, such as the ConcePTION Pregnancy Algorithm [28], with high‐accuracy dating methods where appropriate. Additionally, the potential for machine learning‐based gestational age prediction to perpetuate or exacerbate health disparities remains unexplored. In particular, model performance was not evaluated across maternal race and ethnicity groups, even though race and ethnicity were included as predictors. Although beyond the scope of this study, a systematic evaluation of model fairness, along with the application of bias mitigation techniques, will be essential to ensure transparency and equity. In this context, biased models may systematically misestimate gestational age for certain groups, leading to inaccurate pregnancy onset dating, exposure misclassification, and underrepresentation in pharmacoepidemiologic pregnancy research.

## 5 Conclusion

This study shows that machine‐learning models trained on linked mother–infant EHR data accurately estimated gestational age at delivery overall, with the strongest performance among full‐term and late‐term deliveries and substantially lower performance among preterm deliveries. The novelty of this work lies in leveraging EHR‐derived predictors and mother–infant linkage information within a supervised machine learning framework, demonstrating reproducibility of the modeling approach and portability of the analytic workflow across EHR sites. These findings support further evaluation of the models as tools for retrospectively estimating gestational age in studies with incomplete gestational age data, including direct evaluation in records with missing or invalid gestational age. Continued efforts are needed to improve model performance in estimating gestational age for preterm deliveries and to address and mitigate potential biases.

## Author Contributions

Concept and design: Cosmin A. Bejan, Amelie Pham, Leena Choi, S. Trent Rosenbloom, and Elizabeth J. Phillips. Acquisition, analysis, or interpretation of data: Cosmin A. Bejan, Xiaotong Yang, Amelie Pham, Layth Qassem, Abin Abraham, Leena Choi, S. Trent Rosenbloom, Lana X. Garmire, and Elizabeth J. Phillips. Drafting of the manuscript: Cosmin A. Bejan. Critical review of the manuscript for important intellectual content: all authors. Statistical analysis: Cosmin A. Bejan. Obtained funding: Cosmin A. Bejan and Lana X. Garmire. Administrative, technical, or material support: Xiaotong Yang, Amelie Pham, Layth Qassem, Leena Choi, S. Trent Rosenbloom, Lana X. Garmire, and Elizabeth J. Phillips. Supervision: Cosmin A. Bejan, Lana X. Garmire, and Elizabeth J. Phillips.

## Funding

C.A.B., A.P., L.C., T.R., and E.J.P. are supported by R21HD113234. X.Y. is supported by T32GM141746. L.X.G. is supported by NIH grants R01LM012373, R01LM012907, R03OD039978, and R01HD084633.

## Disclosure

A.P. receives industry‐sponsored research grant funding from the Novocuff medical device company. She also serves as a member of the Data and Safety Monitoring Board (DSMB) for an NIH‐funded study at Vanderbilt University Medical Center (NIH Grant Number: 1R01MH136399). E.J.P. has received consulting fees and royalties from UpToDate, consulting fees from Janssen, AstraZeneca, Intellia, Verve and Glenmark, and grant funding from the NIH (R01HG010863, R01AI152183, U01AI154659, R13AR085978), National Health and Medical Research Council of Australia (NHMRC), and Leo Foundation unrelated to the published work.

## Conflicts of Interest

The authors declare no conflicts of interest.

## Supporting information


**Table S1:** TRIPOD+AI reporting checklist.
**Table S2:** Insufficient prenatal care ICD codes from maternal records.
**Table S3:** Live birth ICD codes from maternal and infant records.
**Table S4:** Text expressions representing valid clinical estimates of gestational age in the EHR.
**Table S5:** Unparseable text expressions for extracting accurate gestational age values in days.
**Table S6:** Hyperparameters used for optimizing the Random Forest and Gradient Boosting models.
**Table S7:** Preterm ICD codes from maternal records.
**Table S8:** Term or postterm ICD codes from maternal records.
**Table S9:** Fetal growth restriction (FGR) ICD codes from maternal records.
**Table S10:** Excessive fetal growth (EFG) ICD codes from maternal records.
**Table S11:** Preterm ICD codes from infant records.
**Table S12:** Term or postterm ICD codes from infant records.
**Table S13:** Small for gestational age (SGA) ICD codes from infant records.
**Table S14:** Large for gestational age (LGA) ICD codes from infant records.
**Table S15:** Overview of predictors for each mother–infant dyad, categorized by data source.
**Table S16:** Algorithm performance results using predictors from maternal records alone or in combination with EHR‐specific data elements.
**Table S17:** Performance results from the temporal stratification of pregnancies delivered before vs. on or after October 1, 2015.
**Table S18:** Evaluation of algorithm performance stratified by delivery status.

## Data Availability

The code used in the analysis is available on GitHub at https://github.com/bejanlab/ML4EGA.git. The summary statistics extracted for this study are provided in the manuscript and Supporting Information S1. Any request to access the Research Derivative data will need to be reviewed and approved by Vanderbilt University Medical Center. Researchers will need to provide evidence of IRB approval for their study. For the approved studies, data will be released via a Data Use Agreement. Interested investigators who meet the criteria for accessing sensitive data can contact the University of Michigan Precision Health's Research Scientific Facilitators at phdatahelp@umich.edu (also see https://research.medicine.umich.edu/our‐units/data‐office‐clinical‐translational‐research/data‐access) to inquire about the UMich dataset and the necessary steps regarding ethics committee approval and data accessing agreement.
