# Supplementary material for: Machine Learning Estimation of Gestational Age at Delivery Using Linked Mother–Infant Electronic Health Records Across Two Health Systems
Source: Pharmacoepidemiol Drug Saf. 2026 Oct 29;35(11):e70516. doi: 10.1002/pds.70516 (PMC13719529; doi:10.1002/pds.70516)
Supplement: Supplementary file 1 [file PDS-35-e70516-s001.docx]

**Machine Learning Estimation of Gestational Age at Delivery Using Linked Mother-Infant Electronic Health Records Across Two Health Systems**

**Supplemental Tables**

**Table S1** TRIPOD+AI reporting checklist.

**Table S2** Insufficient prenatal care ICD codes from maternal records.

**Table S3** Live birth ICD codes from maternal and infant records.

**Table S4** Text expressions representing valid clinical estimates of gestational age in the EHR.

**Table S5** Unparseable text expressions for extracting accurate gestational age values in days.

**Table S6** Hyperparameters used for optimizing the Random Forest and Gradient Boosting models.

**Table S7** Preterm ICD codes from maternal records.

**Table S8** Term or postterm ICD codes from maternal records.

**Table S9** Fetal growth restriction (FGR) ICD codes from maternal records.

**Table S10** Excessive fetal growth (EFG) ICD codes from maternal records.

**Table S11** Preterm ICD codes from infant records.

**Table S12** Term or postterm ICD codes from infant records.

**Table S13** Small for gestational age (SGA) ICD codes from infant records.

**Table S14** Large for gestational age (LGA) ICD codes from infant records.

**Table S15** Overview of predictors for each mother-infant dyad, categorized by data source.

**Table S16** Algorithm performance results using predictors from maternal records alone or in combination with EHR-specific data elements.

**Table S17** Performance results from the temporal stratification of pregnancies delivered before vs. on or after October 1, 2015.

**Table S18** Evaluation of algorithm performance stratified by delivery status.

**Table S1** TRIPOD+AI reporting checklist.

| **Section/Topic** | **Item** | **Development/evaluation** | **Checklist item** | **Reported in the paper** |
| --- | --- | --- | --- | --- |
| **TITLE** | | | | |
| *Title* | 1 | D;E | Identify the study as developing or evaluating the performance of a multivariable prediction model, the target population, and the outcome to be predicted. | *Title* |
| **ABSTRACT** | | | | |
| *Background* | 2.2 | D;E | Provide a brief explanation of the healthcare context and rationale for developing or evaluating the performance of all models. | *Purpose* |
| *Objectives* | 2.3 | D;E | Specify the study objectives, including whether the study describes model development, evaluation, or both. | *Purpose* |
| *Methods* | 2.4 | D;E | Describe the sources of data. | *Methods* |
|  | 2.5 | D;E | Describe the eligibility criteria and setting where the data were collected. | *Methods* |
|  | 2.6 | D;E | Specify the outcome to be predicted by the model, including time horizon of predictions in case of prognostic models. | *Methods* |
|  | 2.7 | D;E | Specify the type of model, a summary of the model-building steps, and the method for internal validation. | *Methods* |
|  | 2.8 | D;E | Specify the measures used to assess model performance (eg, discrimination, calibration, clinical utility). | *Results* |
| *Results* | 2.9 | D;E | Report the number of participants and outcome events. | *Results* |
|  | 2.10 | D;E | Summarize the predictors in the final model. | N/A |
|  | 2.11 | D;E | Report model performance estimates (with confidence intervals). | *Results* |
| *Discussion* | 2.12 | D;E | Give an overall interpretation of the main results. | *Discussion, Conclusion* |
| *Registration* | 2.13 | D;E | Give the registration number and name of the registry or repository. | N/A |
| **INTRODUCTION** | | | | |
| *Background* | 3a | D;E | Explain the healthcare context (including whether diagnostic or prognostic) and rationale for developing or evaluating the prediction model, including references to existing models. | *Introduction* |
|  | 3b | D;E | Describe the target population and the intended purpose of the prediction model in the context of the care pathway, including its intended users (e.g., healthcare professionals, patients, public). | *Introduction* |
|  | 3c | D;E | Describe any known health inequalities between sociodemographic groups. | N/A |
| *Objectives* | 4 | D;E | Specify the study objectives, including whether the study describes the development or validation of a prediction model (or both). | *Introduction* |
| **METHODS** | | | | |
| *Data* | 5a | D;E | Describe the sources of data separately for the development and evaluation datasets (e.g., randomized trial, cohort, routine care or registry data), the rationale for using these data, and representativeness of the data. | *Data sources* |
|  | 5b | D;E | Specify the dates of the collected participant data, including start and end of participant accrual; and, if applicable, end of follow-up. | *Data sources* |
| *Participants* | 6a | D;E | Specify key elements of the study setting (e.g., primary care, secondary care, general population) including the number and location of centres. | *Study population* |
|  | 6b | D;E | Describe the eligibility criteria for study participants. | *Study population* |
|  | 6c | D;E | Give details of any treatments received, and how they were handled during model development or evaluation, if relevant. | N/A |
| *Data preparation* | 7 | D;E | Describe any data pre-processing and quality checking, including whether this was similar across relevant sociodemographic groups. | *EHR-based extraction of gestational age estimates* |
| *Outcome* | 8a | D;E | Clearly define the outcome that is being predicted and the time horizon, including how and when assessed, the rationale for choosing this outcome, and whether the method of outcome assessment is consistent across sociodemographic groups. | *EHR-based extraction of gestational age estimates* |
|  | 8b | D;E | If outcome assessment requires subjective interpretation, describe the qualifications and demographic characteristics of the outcome assessors. | N/A |
|  | 8c | D;E | Report any actions to blind assessment of the outcome to be predicted. | N/A |
| *Predictors* | 9a | D | Describe the choice of initial predictors (e.g., literature, previous models, all available predictors) and any pre-selection of predictors before model building. | *Algorithm-based estimation of gestational age* |
|  | 9b | D;E | Clearly define all predictors, including how and when they were measured (and any actions to blind assessment of predictors for the outcome and other predictors). | *Algorithm-based estimation of gestational age, Table S15* |
|  | 9c | D;E | If predictor measurement requires subjective interpretation, describe the qualifications and demographic characteristics of the predictor assessors. | N/A |
| *Sample size* | 10 | D;E | Explain how the study size was arrived at (separately for development and evaluation), and justify that the study size was sufficient to answer the research question. Include details of any sample size calculation. | *Algorithm-based estimation of gestational age* |
| *Missing data* | 11 | D;E | Describe how missing data were handled. Provide reasons for omitting any data. | *Algorithm-based estimation of gestational age* |
| *Analytical methods* | 12a | D | Describe how the data were used (e.g., for development and evaluation of model performance) in the analysis, including whether the data were partitioned, considering any sample size requirements. | *Algorithm-based estimation of gestational age* |
|  | 12b | D | Depending on the type of model, describe how predictors were handled in the analyses (functional form, rescaling, transformation, or any standardisation). | *Algorithm-based estimation of gestational age* |
|  | 12c | D | Specify the type of model, rationale, all model-building steps, including any hyperparameter tuning, and method for internal validation. | *Algorithm-based estimation of gestational age* |
|  | 12d | D;E | Describe if and how any heterogeneity in estimates of model parameter values and model performance was handled and quantified across clusters (e.g., hospitals, countries). See TRIPOD-Cluster for additional considerations. | *Evaluation* |
|  | 12e | D;E | Specify all measures and plots used (and their rationale) to evaluate model performance (e.g., discrimination, calibration, clinical utility) and, if relevant, to compare multiple models. | *Evaluation* |
|  | 12f | E | Describe any model updating (e.g., recalibration) arising from the model evaluation, either overall or for particular sociodemographic groups or settings. | *Evaluation* |
|  | 12g | E | For model evaluation, describe how the model predictions were calculated (e.g., formula, code, object, application programming interface). | *Algorithm-based estimation of gestational age* |
| *Class imbalance* | 13 | D;E | If class imbalance methods were used, state why and how this was done, and any subsequent methods to recalibrate the model or the model predictions. | N/A |
| *Fairness* | 14 | D;E | Describe any approaches that were used to address model fairness and their rationale. | N/A |
| *Model output* | 15 |  | Specify the output of the prediction model (e.g., probabilities, classification). Provide details and rationale for any classification and how the thresholds were identified. | *Algorithm-based estimation of gestational age* |
| *Training versus evaluation* | 16 | D;E | Identify any differences between the development and evaluation data in healthcare setting, eligibility criteria, outcome, and predictors. | *Algorithm-based estimation of gestational age, Method replication* |
| *Ethical approval* | 17 | D;E | Name the institutional research board or ethics committee that approved the study and describe the participant-informed consent or the ethics committee waiver of informed consent. | *Methods* |
| **OPEN SCIENCE** | | | | |
| *Funding* | 18a | D;E | Give the source of funding and the role of the funders for the present study. | *Funding* |
| *Conflicts of interest* | 18b | D;E | Declare any conflicts of interest and financial disclosures for all authors. | *Disclosures* |
| *Protocol* | 18c | D;E | Indicate where the study protocol can be accessed or state that a protocol was not prepared. | N/A |
| *Registration* | 18d | D;E | Provide registration information for the study, including register name and registration number, or state that the study was not registered. | N/A |
| *Data sharing* | 18e | D;E | Provide details of the availability of the study data. | *Data availability* |
| *Code sharing* | 18f | D;E | Provide details of the availability of the analytical code. | *Data availability* |
| **PATIENT & PUBLIC INVOLVEMENT** | | | | |
| *Patient & Public Involvement* | 19 | D;E | Provide details of any patient and public involvement during the design, conduct, reporting, interpretation, or dissemination of the study or state no involvement. | N/A |
| **RESULTS** | | | | |
| *Participants* | 20a | D;E | Describe the flow of participants through the study, including the number of participants with and without the outcome and, if applicable, a summary of the follow-up time. A diagram may be helpful. | *Selection of mother-infant dyads, Figure 1* |
|  | 20b | D;E | Report the characteristics overall and, where applicable, for each data source or setting, including the key dates, key predictors (including demographics), treatments received, sample size, number of outcome events, follow-up time, and amount of missing data. A table may be helpful. Report any differences across key demographic groups. | *Selection of mother-infant dyads, Table 1* |
|  | 20c | E | For model evaluation, show a comparison with the development data of the distribution of important predictors (demographics, predictors, and outcome). | *Predictor analysis in association with CGA, Figure 2* |
| *Model development* | 21 | D;E | Specify the number of participants and outcome events in each analysis (e.g., for model development, hyperparameter tuning, model evaluation). | *CGA estimation performance, Tables 2, S16-18* |
| *Model specification* | 22 | D | Provide details of the full prediction model (e.g., formula, code, object, application programming interface) to allow predictions in new individuals and to enable third-party evaluation and implementation, including any restrictions to access or re-use (e.g., freely available, proprietary). | *Data availability* |
| *Model performance* | 23a | D;E | Report model performance estimates with confidence intervals, including for any key subgroups (e.g., sociodemographic). Consider plots to aid presentation. | *CGA estimation performance, Figure 3, Tables 2, S16-18* |
|  | 23b | D;E | If examined, report results of any heterogeneity in model performance across clusters. See TRIPOD Cluster for additional details. | *CGA estimation performance, Figure 3, Tables S16-18* |
| *Model updating* | 24 | E | Report the results from any model updating, including the updated model and subsequent performance. | N/A |
| **DISCUSSION** | | | | |
| *Interpretation* | 25 | D;E | Give an overall interpretation of the main results, including issues of fairness in the context of the objectives and previous studies. | *Discussion* |
| *Limitations* | 26 | D;E | Discuss any limitations of the study (such as a non-representative sample, sample size, overfitting, missing data) and their effects on any biases, statistical uncertainty, and generalizability. | *Discussion* |
| *Usability of the model in the context of current care* | 27a | D | Describe how poor quality or unavailable input data (e.g., predictor values) should be assessed and handled when implementing the prediction model. | *Discussion* |
|  | 27b | D | Specify whether users will be required to interact in the handling of the input data or use of the model, and what level of expertise is required of users. | N/A |
|  | 27c | D;E | Discuss any next steps for future research, with a specific view to applicability and generalizability of the model. | *Discussion, Conclusion* |
| D=items relevant only to the development of a prediction model; E=items relating solely to the evaluation of a prediction model; D;E=items applicable to both the development and evaluation of a prediction model. | | | | |

**Table S2** Insufficient prenatal care ICD codes from maternal records.

| Code | Description |
| --- | --- |
| ICD-9-CM |  |
| V23.7 | Supervision of high-risk pregnancy with insufficient prenatal care |
|  |  |
| ICD-10-CM |  |
| O09.3xx | Supervision of pregnancy with insufficient antenatal care |

**Table S3** Live birth ICD codes from maternal and infant records.

| Code | Description |
| --- | --- |
| ICD-9-CM |  |
| 765.1x | Other preterm infants |
| 766.0 | Exceptionally large baby |
| 766.1 | Other "heavy-for-dates" infants |
| 766.2x | Late infant |
| V27.0 | Outcome of delivery, single liveborn |
| V27.2 | Outcome of delivery, twins, both liveborn |
| V27.5 | Outcome of delivery, other multiple birth, all liveborn |
| V30.xx | Single liveborn |
| V31.xx | Twin, mate liveborn |
| V34.xx | Other multiple, mates all liveborn |
| V39.xx | Liveborn, unspecified whether single, twin or multiple |
|  |  |
| ICD-10-CM |  |
| P07.0x | Extremely low birth weight newborn |
| P07.1x | Other low birth weight newborn |
| P07.3x | Preterm [premature] newborn |
| P08.0 | Exceptionally large newborn baby |
| P08.1 | Other heavy for gestational age newborn |
| P08.2 | Late newborn, not heavy for gestational age |
| P08.21 | Post-term newborn |
| P08.22 | Prolonged gestation of newborn |
| Z37.0 | Single live birth |
| Z37.2 | Twins, both liveborn |
| Z37.5x | Other multiple births, all liveborn |
| Z38.xx | Single or multiple liveborn infants according to place of birth and type of delivery |

**Table S4** Text expressions representing valid clinical estimates of gestational age in the EHR.

| 40 weeks | 36.5 | 28 weeks 3 days | 38 weeks 2/7 | 41wks |
| --- | --- | --- | --- | --- |
| 40 4/7 weeks | 36w2d | 37/5 | 36.5 weeks EGA | 40 wks. |
| 38w 2d | 35 5/7 days | 41 3/7wks | 38 2/7 weeks2 | 42weeks |
| 40 4/7 | 37 4/7weeks | 37.4 wks | 29 + 6weeks | 40weeks + 2 days |
| 40 | 37.4weeks | 34 weeks 0/7 days | 32 and 4/7 weeks | 40 weeks 1 day |
| 39w | 37weeks | 39 wks | 41 weeks & 1 day | 40 weeks and 2 days |
| 44 4/7 wks | 37+2 | 27weeks 1day | 39wks 5days | 41/1weeks |
| 41.1 weeks | 39/1 weeks | 40+1 weeks | 41 and 4 weeks | 39 1 /7 weeks |

**Table S5** Unparseable text expressions for extracting accurate gestational age values in days.

| 36-39 | Delivered | ~ 35 | ~42 weeks |
| --- | --- | --- | --- |
| 33-35 wks | no dating | 10/7/09 | 41 60/7 weeks |
| PP | unknown, term | unknown, est 34-36 by pt | approx 32 weeks |

**Table S6** Hyperparameters used for optimizing the random forest and gradient boosting models.

| **Model hyperparameter** | | **Value range** | **Optimal values on VUMC dataset** | **Optimal values on UMich dataset** |
| --- | --- | --- | --- | --- |
| **Random Forest** | |  |  |  |
|  | n_estimators | [100, 200] | 200 | 200 |
|  | max_depth | [None, 10, 20] | None | None |
|  | min_samples_split | [2, 5] | 5 | 2 |
|  | min_samples_leaf | [1, 2] | 1 | 1 |
|  | max_features | ['sqrt', 'log2'] | sqrt | sqrt |
| **Gradient Boosting** | |  |  |  |
|  | n_estimators | [100, 200] | 200 | 200 |
|  | learning_rate | [0.01, 0.1, 0.2] | 0.2 | 0.2 |
|  | max_depth | [3, 5, 7] | 7 | 7 |
|  | min_samples_split | [2, 5] | 5 | 2 |
|  | min_samples_leaf | [1, 3] | 1 | 3 |
|  | subsample | [0.8, 1.0] | 1.0 | 1.0 |
|  | max_features | ['sqrt', 'log2'] | sqrt | sqrt |

**Table S7** Preterm ICD codes from maternal records.

| Code | Description |
| --- | --- |
| ICD-9-CM |  |
| 644.2x | Early onset of delivery |
|  |  |
| ICD-10-CM |  |
| O42.01x | Preterm premature rupture of membranes, onset of labor within 24 hours of rupture |
| O42.11x | Preterm premature rupture of membranes, onset of labor more than 24 hours following rupture |
| O42.91x | Preterm premature rupture of membranes, unspecified as to length of time between rupture and onset of labor |
| O60.1 | Preterm labor with preterm delivery |
| O60.10xx | Preterm labor with preterm delivery, unspecified trimester |
| O60.12xx | Preterm labor second trimester with preterm delivery second trimester |
| O60.13xx | Preterm labor second trimester with preterm delivery third trimester |
| O60.14xx | Preterm labor third trimester with preterm delivery third trimester |

**Table S8** Term or postterm ICD codes from maternal records.

| Code | Description |
| --- | --- |
| ICD-9-CM |  |
| 645 | Late pregnancy |
| 645.0x | Prolonged pregnancy |
| 645.1x | Post term pregnancy |
| 645.2x | Prolonged pregnancy |
|  |  |
| ICD-10-CM |  |
| O42.92 | Full-term premature rupture of membranes, unspecified as to length of time between rupture and onset of labor |
| O48 | Late pregnancy |
| O48.0 | Post-term pregnancy |
| O48.1 | Prolonged pregnancy |
| O60.20Xx | Term delivery with preterm labor, unspecified trimester |
| O60.22Xx | Term delivery with preterm labor, second trimester |
| O60.23Xx | Term delivery with preterm labor, third trimester |
| O80 | Encounter for full-term uncomplicated delivery |
| Z3A.37 | 37 weeks gestation of pregnancy |
| Z3A.38 | 38 weeks gestation of pregnancy |
| Z3A.39 | 39 weeks gestation of pregnancy |
| Z3A.4 | Weeks of gestation of pregnancy, weeks 40 or greater |
| Z3A.40 | 40 weeks gestation of pregnancy |
| Z3A.41 | 41 weeks gestation of pregnancy |
| Z3A.42 | 42 weeks gestation of pregnancy |
| Z3A.49 | Greater than 42 weeks gestation of pregnancy |

**Table S9** Fetal growth restriction (FGR) ICD codes from maternal records.

| Code | Description |
| --- | --- |
| ICD-9-CM |  |
| 656.5x | Poor fetal growth affecting management of mother |
| V28.4 | Antenatal screening for fetal growth retardation using ultrasonics |
|  |  |
| ICD-10-CM |  |
| O36.5 | Maternal care for known or suspected poor fetal growth |
| O36.51 | Maternal care for known or suspected placental insufficiency |
| O36.511x | Maternal care for known or suspected placental insufficiency, first trimester |
| O36.512x | Maternal care for known or suspected placental insufficiency, second trimester |
| O36.513x | Maternal care for known or suspected placental insufficiency, third trimester |
| O36.519x | Maternal care for known or suspected placental insufficiency, unspecified trimester |
| O36.59 | Maternal care for other known or suspected poor fetal growth |
| O36.591x | Maternal care for other known or suspected poor fetal growth, first trimester |
| O36.592x | Maternal care for other known or suspected poor fetal growth, second trimester |
| O36.593x | Maternal care for other known or suspected poor fetal growth, third trimester |
| O36.599x | Maternal care for other known or suspected poor fetal growth, unspecified trimester |
| Z36.4 | Encounter for antenatal screening for fetal growth retardation |

**Table S10** Excessive fetal growth (EFG) ICD codes from maternal records.

| Code | Description |
| --- | --- |
| ICD-9-CM |  |
| 656.6x | Excessive fetal growth affecting management of mother |
|  |  |
| ICD-10-CM |  |
| O36.60xx | Maternal care for excessive fetal growth, unspecified trimester |
| O36.61xx | Maternal care for excessive fetal growth, first trimester |
| O36.62xx | Maternal care for excessive fetal growth, second trimester |
| O36.63xx | Maternal care for excessive fetal growth, third trimester |
| Z36.88 | Encounter for antenatal screening for fetal macrosomia |

**Table S11** Preterm ICD codes from infant records.

| Code | Description |
| --- | --- |
| ICD-9-CM |  |
| 765 | Disorders relating to short gestation and unspecified low birthweight |
| 765.0x | Extreme immaturity |
| 765.1x | Other preterm infants |
| 774.2 | Neonatal jaundice associated with preterm delivery |
|  |  |
| ICD-10-CM |  |
| P07 | Disorders of newborn related to short gestation and low birth weight, not elsewhere classified |
| P07.0x | Extremely low birth weight newborn |
| P07.1x | Other low birth weight newborn |
| P07.2x | Extreme immaturity of newborn |
| P07.3x | Preterm [premature] newborn [other] |
| P59.0 | Neonatal jaundice associated with preterm delivery |

**Table S12** Term or postterm ICD codes from infant records.

| Code | Description |
| --- | --- |
| ICD-9-CM |  |
| 765.29 | 37 or more completed weeks of gestation |
| 766.2 | Late infant, not "heavy-for-dates" |
| 766.21 | Post-term infant |
| 766.22 | Prolonged gestation of infant |
|  |  |
| ICD-10-CM |  |
| P08.2 | Late newborn, not heavy for gestational age |
| P08.21 | Post-term newborn |
| P08.22 | Prolonged gestation of newborn |

**Table S13** Small for gestational age (SGA) ICD codes from infant records.

| Code | Description |
| --- | --- |
| ICD-9-CM |  |
| 764 | Slow fetal growth and fetal malnutrition |
| 764.0x | ‘Light-for-dates’ without mention of fetal malnutrition |
| 764.1x | ‘Light-for-dates’ with signs of fetal malnutrition |
| 764.2x | Fetal malnutrition without mention of ‘light-for-dates’ |
| 764.9x | Fetal growth retardation, unspecified |
|  |  |
| ICD-10-CM |  |
| P05 | Disorders of newborn related to slow fetal growth and fetal malnutrition |
| P05.0x | Newborn light for gestational age |
| P05.1x | Newborn small for gestational age |
| P05.2 | Newborn affected by fetal (intrauterine) malnutrition not light or small for gestational age |
| P05.9 | Newborn affected by slow intrauterine growth, unspecified |

**Table S14** Large for gestational age (LGA) ICD codes from infant records.

| Code | Description |
| --- | --- |
| ICD-9-CM |  |
| 766 | Disorders relating to long gestation and high birthweight |
| 766.0 | Exceptionally large baby |
| 766.1 | Other "heavy-for-dates" infants |
|  |  |
| ICD-10-CM |  |
| P08 | Disorders of newborn related to long gestation and high birth weight |
| P08.0 | Exceptionally large newborn baby |
| P08.1 | Other heavy for gestational age newborn |

**Table S15** Overview of predictors for each mother-infant dyad, categorized by data source.

| **Predictor category** | **Description** |
| --- | --- |
| **Maternal** | Maternal age at delivery |
|  | Maternal race and ethnicity |
|  | Preterm ICD codes from maternal records |
|  | Term or postterm ICD codes from maternal records |
|  | Fetal growth restriction ICD codes from maternal records |
|  | Excessive fetal growth ICD codes from maternal records |
| **EHR** | Number of previous pregnancies |
|  | Number of fetuses in the current pregnancy |
|  | The APGAR score at 1 minute after birth from maternal records |
|  | The APGAR score at 5 minutes after birth from maternal records |
|  | Infant weight at birth from maternal records |
| **Pediatric** | Infant sex |
|  | Infant race and ethnicity |
|  | Preterm ICD codes from infant records |
|  | Term or postterm ICD codes from infant records |
|  | Small for gestational age ICD codes from infant records |
|  | Large for gestational age ICD codes from infant records |

**Table S16** Algorithm performance results using predictors from maternal records alone or in combination with EHR-specific data elements.

| **Dataset** | **Dyads** | **Predictor**  **category** | **Algorithm** | **% within**  **1 week (95% CI)** | **% within**  **2 weeks (95% CI)** | **MAE (95% CI)** |
| --- | --- | --- | --- | --- | --- | --- |
| VUMC | 10,869 | Maternal | Random Forest | 82.1 (81.4-82.8) | 91.6 (91.1-92.1) | 5.2 (5.0-5.4) |
|  |  |  | Gradient Boosting | 81.1 (80.3-81.8) | 91.4 (90.8-91.9) | 5.5 (5.3-5.7) |
|  |  |  | Ensemble | 82.3 (81.6-83.0) | 91.9 (91.4-92.4) | 5.2 (5.0-5.4) |
|  |  | Maternal + EHR | Random Forest | 83.1 (82.4-83.8) | 92.6 (92.1-93.1) | 4.7 (4.6-4.9) |
|  |  |  | Gradient Boosting | 82.3 (81.6-83.1) | 92.5 (92.1-93.0) | 4.9 (4.8-5.1) |
|  |  |  | Ensemble | 83.2 (82.5-83.9) | 93.1 (92.6-93.6) | 4.7 (4.6-4.9) |
| UMich | 6,869 | Maternal | Random Forest | 88.1 (87.3-88.8) | 94.1 (93.5-94.6) | 4.1 (3.9-4.3) |
|  |  |  | Gradient Boosting | 86.9 (86.1-87.6) | 93.8 (93.2-94.3) | 4.2 (4.1-4.4) |
|  |  |  | Ensemble | 87.8 (87.0-88.5) | 94.2 (93.7-94.7) | 4.0 (3.9-4.2) |
|  |  | Maternal + EHR | Random Forest | 90.6 (89.9-91.2) | 96.2 (95.8-96.7) | 3.2 (3.1-3.3) |
|  |  |  | Gradient Boosting | 89.7 (89.0-90.4) | 96.4 (96.0-96.8) | 3.2 (3.1-3.3) |
|  |  |  | Ensemble | 90.9 (90.2-91.5) | 96.8 (96.4-97.2) | 3.1 (3.0-3.2) |
| MAE, mean absolute error; CI, confidence interval | | | | | | |

**Table S17** Performance results from the temporal stratification of pregnancies delivered before vs. on or after October 1, 2015.

| **Dataset** | **Delivery date** | **Dyads** | **Algorithm** | **% within**  **1 week (95% CI)** | **% within**  **2 weeks (95% CI)** | **MAE (95% CI)** |
| --- | --- | --- | --- | --- | --- | --- |
| VUMC | Before 10/01/2015 | 3,454 | Random Forest | 77.6 (76.3-79.0) | 95.3 (94.6-96.0) | 5.1 (4.9-5.3) |
|  |  | (31.8%) | Gradient Boosting | 76.5 (75.1-78.0) | 96.2 (95.5-96.8) | 5.0 (4.9-5.2) |
|  |  |  | Ensemble | 77.6 (76.2-79.1) | 96.2 (95.6-96.8) | 4.9 (4.7-5.1) |
|  | On/after 10/01/2015 | 7,415 | Random Forest | 88.7 (88.0-89.3) | 93.3 (92.7-93.9) | 4.0 (3.8-4.2) |
|  |  | (68.2%) | Gradient Boosting | 87.4 (86.7-88.2) | 92.6 (92.0-93.1) | 4.5 (4.2-4.7) |
|  |  |  | Ensemble | 88.6 (87.9-89.3) | 93.4 (92.8-93.9) | 4.1 (3.9-4.3) |
| UMich | Before 10/01/2015 | 828 | Random Forest | 79.6 (76.7-82.4) | 96.9 (95.5-97.9) | 4.7 (4.4-5.1) |
|  |  | (12.1%) | Gradient Boosting | 79.9 (77.3-82.5) | 97.1 (95.9-98.2) | 4.7 (4.4-5.0) |
|  |  |  | Ensemble | 80.9 (78.3-83.5) | 97.4 (96.4-98.6) | 4.6 (4.3-4.9) |
|  | On/after 10/01/2015 | 6,041 | Random Forest | 94.7 (94.1-95.2) | 97.6 (97.2-98.0) | 2.6 (2.5-2.7) |
|  |  | (87.9%) | Gradient Boosting | 92.7 (92.0-93.3) | 97.0 (96.6-97.5) | 2.9 (2.8-3.0) |
|  |  |  | Ensemble | 94.8 (94.2-95.4) | 97.9 (97.5-98.2) | 2.5 (2.4-2.6) |
| MAE, mean absolute error; CI, confidence interval | | | | | | |

**Table S18** Evaluation of algorithm performance stratified by delivery status.

| **Dataset** | **Delivery**  **status** | **Dyads**  **N (%)** | **Algorithm** | **% within**  **1 week (95% CI)** | **% within**  **2 weeks (95% CI)** | **MAE (95% CI)** |
| --- | --- | --- | --- | --- | --- | --- |
| VUMC | Preterm | 1,569 | Random Forest | 39.5 (36.9-41.9) | 64.9 (62.5-67.4) | 14.5 (13.7-15.2) |
|  |  | (14.4%) | Gradient Boosting | 33.9 (31.5-36.4) | 60.3 (58.0-62.7) | 16.2 (15.4-17.0) |
|  |  |  | Ensemble | 39.8 (37.3-42.1) | 65.1 (62.8-67.5) | 14.6 (13.9-15.4) |
|  | Early term | 2,839 | Random Forest | 87.6 (86.3-88.7) | 98.7 (98.2-99.1) | 3.4 (3.2-3.5) |
|  |  | (26.1%) | Gradient Boosting | 88.5 (87.3-89.6) | 99.2 (98.8-99.5) | 3.4 (3.3-3.5) |
|  |  |  | Ensemble | 88.3 (87.1-89.4) | 99.1 (98.8-99.5) | 3.3 (3.2-3.4) |
|  | Full term | 5,551 | Random Forest | 94.8 (94.2-95.3) | 98.8 (98.5-99.1) | 2.4 (2.4-2.5) |
|  |  | (51.1%) | Gradient Boosting | 93.8 (93.2-94.5) | 99.4 (99.2-99.6) | 2.5 (2.4-2.5) |
|  |  |  | Ensemble | 94.3 (93.7-94.9) | 99.2 (99.0-99.4) | 2.4 (2.3-2.5) |
|  | Late term | 866 | Random Forest | 98.1 (97.2-99.0) | 99.7 (99.2-100.0) | 1.7 (1.6-1.8) |
|  |  | (8.0%) | Gradient Boosting | 96.6 (95.4-97.8) | 99.4 (98.8-99.9) | 1.8 (1.6-1.9) |
|  |  |  | Ensemble | 97.8 (96.8-98.7) | 99.5 (99.1-99.9) | 1.7 (1.6-1.9) |
|  | Postterm | 44 | Random Forest | 86.2 (75.0-95.5) | 100.0 (100.0-100.0) | 4.9 (4.2-5.7) |
|  |  | (0.4%) | Gradient Boosting | 81.7 (70.5-93.2) | 97.6 (93.2-100.0) | 4.8 (3.8-5.8) |
|  |  |  | Ensemble | 83.8 (72.7-93.2) | 100.0 (100.0-100.0) | 4.5 (3.6-5.4) |
| UMich | Preterm | 901 | Random Forest | 64.2 (61.0-67.4) | 83.0 (80.6-85.6) | 7.8 (7.2-8.4) |
|  |  | (13.1%) | Gradient Boosting | 54.8 (51.6-58.0) | 79.0 (76.4-81.6) | 8.9 (8.4-9.5) |
|  |  |  | Ensemble | 66.4 (63.4-69.4) | 84.5 (82.1-86.7) | 7.4 (6.9-8.0) |
|  | Early term | 1,734 | Random Forest | 94.8 (93.8-95.8) | 99.4 (99.0-99.7) | 2.5 (2.4-2.6) |
|  |  | (25.2%) | Gradient Boosting | 93.7 (92.5-94.8) | 99.4 (99.0-99.8) | 2.9 (2.8-3.0) |
|  |  |  | Ensemble | 95.0 (94.0-96.0) | 99.6 (99.3-99.9) | 2.6 (2.5-2.7) |
|  | Full term | 3,636 | Random Forest | 98.1 (97.6-98.5) | 99.9 (99.7-100.0) | 1.9 (1.9-2.0) |
|  |  | (52.9%) | Gradient Boosting | 97.8 (97.3-98.3) | 99.9 (99.8-100.0) | 2.0 (1.9-2.0) |
|  |  |  | Ensemble | 97.9 (97.4-98.4) | 99.9 (99.8-100.0) | 1.9 (1.9-2.0) |
|  | Late term | 572 | Random Forest | 99.5 (98.8-100.0) | 100.0 (100.0-100.0) | 1.4 (1.3-1.6) |
|  |  | (8.3%) | Gradient Boosting | 98.9 (98.1-99.7) | 100.0 (100.0-100.0) | 1.4 (1.3-1.6) |
|  |  |  | Ensemble | 99.5 (98.8-100.0) | 100.0 (100.0-100.0) | 1.4 (1.3-1.5) |
|  | Postterm | 26 | Random Forest | 92.3 (80.8-100.0) | 100.0 (100.0-100.0) | 3.8 (2.9-4.8) |
|  |  | (0.4%) | Gradient Boosting | 80.9 (65.4-96.2) | 96.2 (88.5-100.0) | 3.6 (2.3-5.1) |
|  |  |  | Ensemble | 92.3 (80.8-100.0) | 100.0 (100.0-100.0) | 2.7 (1.7-3.9) |
| MAE, mean absolute error; CI, confidence interval | | | | | | |
